# *n*-Alcohol Length Governs Shift in *L*_o_-*L*_d_ Mixing Temperatures in Synthetic and Cell-Derived Membranes

**DOI:** 10.1016/j.bpj.2017.06.066

**Published:** 2017-08-09

**Authors:** Caitlin E. Cornell, Nicola L.C. McCarthy, Kandice R. Levental, Ilya Levental, Nicholas J. Brooks, Sarah L. Keller

**Affiliations:** 1 University of Washington, Department of Chemistry, Seattle, Washington; 2 Department of Chemistry, Imperial College London, London, United Kingdom; 3 Department of Integrative Biology and Pharmacology, McGovern Medical School at The University of Texas Medical Center, Houston, Texas

## Abstract

A persistent challenge in membrane biophysics has been to quantitatively predict how membrane physical properties change upon addition of new amphiphiles (e.g., lipids, alcohols, peptides, or proteins) in order to assess whether the changes are large enough to plausibly result in biological ramifications. Because of their roles as general anesthetics, *n*-alcohols are perhaps the best-studied amphiphiles of this class. When *n*-alcohols are added to model and cell membranes, changes in membrane parameters tend to be modest. One striking exception is found in the large decrease in liquid-liquid miscibility transition temperatures (*T*_mix_) observed when short-chain *n*-alcohols are incorporated into giant plasma membrane vesicles (GPMVs). Coexisting liquid-ordered and liquid-disordered phases are observed at temperatures below *T*_mix_ in GPMVs as well as in giant unilamellar vesicles (GUVs) composed of ternary mixtures of a lipid with a low melting temperature, a lipid with a high melting temperature, and cholesterol. Here, we find that when GUVs of canonical ternary mixtures are formed in aqueous solutions of short-chain *n*-alcohols (n ≤ 10), *T*_mix_ increases relative to GUVs in water. This shift is in the opposite direction from that reported for cell-derived GPMVs. The increase in *T*_mix_ is robust across GUVs of several types of lipids, ratios of lipids, types of short-chain *n*-alcohols, and concentrations of *n*-alcohols. However, as chain lengths of *n*-alcohols increase, nonmonotonic shifts in *T*_mix_ are observed. Alcohols with chain lengths of 10–14 carbons decrease *T*_mix_ in ternary GUVs of dioleoyl-PC/dipalmitoyl-PC/cholesterol, whereas 16 carbons increase *T*_mix_ again. Gray et al. observed a similar influence of the length of *n*-alcohols on the direction of the shift in *T*_mix_. These results are consistent with a scenario in which the relative partitioning of *n*-alcohols between liquid-ordered and liquid-disordered phases evolves as the chain length of the *n*-alcohol increases.

## Introduction

Scientists have invested decades of research into understanding how *n*-alcohols affect model lipid membranes, largely with the goal of clarifying mechanisms by which ethanol consumption perturbs mammalian cell membranes. The results tell a compelling story: *n*-alcohols partition into membranes near lipid headgroups where they disorder carbon chains of neighboring lipids or probes (1, 2, 3). Concomitantly, *n*-alcohols alter physical properties of liquid-phase membranes: lipid lateral mobilities increase (4, 5, 6), ion channel cation permeabilities increase (7), membrane areas increase (8), thicknesses decrease (9), bending moduli decrease (8), area compressibilities decrease (8), interfacial tensions decrease (8), gel-liquid transition temperatures decrease (10), *L*_α_-*H*_II_ transition temperatures shift (11), and lateral pressure profiles shift (6, 12). However, the magnitudes of most of these effects are modest. For example, relatively high concentrations of ethanol (120 mM) decrease membrane gel-liquid melting temperatures by only 0.3°C (10).

One striking exception to the rule that *n*-alcohols tend to have a minimal effect on physical properties of membranes was recently reported by Veatch and colleagues (13). Using cell-derived giant plasma membrane vesicles (GPMVs), they found that short-chain *n*-alcohols dramatically decreased miscibility transition temperatures (*T*_mix_). The shift in *T*_mix_ (∼4°C for 120 mM ethanol) is more than an order of magnitude larger than ethanol’s effect on membrane melting temperatures (10, 13). 120 mM ethanol is the concentration reported by Pringle et al. (14) as the anesthetic concentration (AC50) at which 50% of tadpoles lose their righting reflex. The ethanol concentration at which proteins begin to denature is at least an order of magnitude higher (15). Strikingly, the result that short-chain *n*-alcohols decrease *T*_mix_ of GPMVs by ∼4°C holds equally well for ethanol, propanol, octanol, and decanol at the AC50 concentration (13).

Cell-derived GPMVs have several advantages as experimental systems. They are large enough (∼10 *μ*m) to image by conventional microscopy; they contain significant amounts of functioning, native proteins; they retain extraordinary complexity in their lipid and protein compositions (similar to cell plasma membranes); and the spatial distribution of the lipids and proteins in their membranes can be probed by fluorophores (16, 17, 18, 19, 20, 21). At high temperatures, GPMV membranes appear uniform by epifluorescence microscopy. Below *T*_mix_, GPMV lipids and proteins demix into coexisting liquid-ordered (*L*_o_) and liquid-disordered (*L*_d_) phases (18, 19, 20, 21).

The result that *n*-alcohols dramatically shift miscibility transition temperatures in GPMVs leads to the clear question of whether *n*-alcohols also shift *T*_mix_ in simpler membranes of giant unilamellar vesicles (GUVs) composed of ternary lipid mixtures. We are motivated to ask this question because the phenomenon of membranes demixing into *L*_o_ and *L*_d_ phases has been largely understood in the context of GUVs composed of ternary mixtures of a lipid with a high melting temperature, a lipid with a low melting temperature, and cholesterol. The relative amounts of each lipid type can be quantitatively tuned in GUVs (22), making them an ideal system for mapping phase diagrams. General features within phase diagrams of ternary membranes are relatively well understood. For example, researchers know how to tune lipid ratios to achieve membranes that are likely to exhibit gel phases, coexisting *L*_o_ and *L*_d_ phases, or critical phenomena, and they have used this information to provide a broader context to interpret results from specific cell-derived membranes (21, 23, 24). However, an enduring challenge has been to quantitatively predict the effect of substituting or adding new membrane components (19, 25), including *n*-alcohols.

Here, we find that the addition of short-chain *n*-alcohols to ternary GUVs significantly shifts miscibility transition temperatures, and that the magnitude of the shift is large, as in cell-derived GPMVs. However, to our surprise, we find that the direction of the shift is opposite in the two systems. We describe experiments that explore this phenomenon, and we offer a plausible speculation to explain why short-chain *n*-alcohols decrease miscibility transition temperatures in GPMVs and increase them in GUVs.

## Materials and Methods

### Materials

Phosphocholine (PC) lipids including DOPC (dioleoyl-PC, or di-18:1-PC), DPPC (dipalmitoyl-PC or di-16:0-PC), POPC (palmitoyl-oleoyl-PC or 16:0-18:1-PC), di16:1-Δ9cis-PC, di18:1-Δ6cis-PC, di14:1-Δ9cis-PC, lyso(18:0)-PC, and palmitoyl sphingomyelin (PSM or 18:1-16:0 SM) were from Avanti Polar Lipids (Alabaster, AL). Texas Red dihexadecanoyl-phosphoethanolamine was from Life Technologies (Grand Island, NY), and cholesterol was from Sigma Aldrich (St. Louis, MO). Lipid structures appear in Fig. S1. Stock solutions of laurdan (Invitrogen, Carlsbad, CA) and C-laurdan (a gift from B. R. Cho, Seoul, Korea) were prepared in ethanol and dimethyl sulfoxide (DMSO). Alcohols (ethanol, propanol, butanol, pentanol, hexanol, octanol, decanol, tetradecanol, hexadecanol, propofol, and 2,6-di-*tert*-butylphenol), DMSO, and all additional reagents were from Sigma Aldrich unless specified. All alcohols were purchased at their highest available purity. All materials were used as from the manufacturer without further purification.

### Production of GUVs for *T*_mix_ measurements

GUVs with diameters in the order of 10^1^–10^2^ *μ*m were electroformed (26) in either pure (18 MΩ-cm) water or in alcohol solutions (0.0025–480 mM alcohol in 18 MΩ-cm water). At ethanol concentrations above 1.2 M, PC-membranes are partially solubilized; above 7 M, mixed micelles form (27). Alcohols with low water solubilities (tetradecanol, hexadecanol, and 2,6-di-*tert*-butylphenol) were first dissolved in DMSO before being dissolved in water. For these alcohols, the maximum final concentration of DMSO in aqueous solution was 210 mM, too small to measurably affect miscibility transition temperatures. Control experiments were conducted to verify that shifts in *T*_mix_ values due to producing GUVs of 35:35:30 DOPC/DPPC/cholesterol in 18 MΩ-cm water (*T*_mix_ = 30.4 ± 0.29°C) versus in DMSO solutions ∼7-fold more concentrated (1.4M) than those used in *n*-alcohol experiments (*T*_mix_ = 31.1 ± 0.04°C) were smaller than shifts in *T*_mix_ due to the type of *n*-alcohol used.

GUVs used in measurements of *T*_mix_ were electroformed as follows. An aliquot of 0.25 mg of lipids in chloroform was spread evenly on an indium-tin-oxide-coated glass slide (Delta Technologies, Loveland, CO). The lipid mixture contained 0.8 mol% Texas Red dihexadecanoyl-phosphoethanolamine, a dye-labeled lipid that selectively partitions to the *L*_d_ phase (28). The slide was placed under vacuum for > 30 min to evaporate the chloroform. A capacitor was created by separating two indium-tin-oxide-coated slides with two rectangular Teflon bars (0.3mm thick). The gap between the bars was filled with water or alcohol solution, and all edges were sealed with vacuum grease. An AC voltage of 10 Hz and 1.5 V was applied to the capacitor for 1 h at 60°C. Vesicles were then extracted from the capacitor and diluted 5–10-fold in water or alcohol solution at 60°C to make a stock solution.

### Measurement of *T*_mix_

Electroformation produces populations of vesicles with distributions of miscibility transition temperatures, reflecting slight differences in lipid ratios from vesicle to vesicle. Uncertainties in ratios of PC-lipids in electroformed vesicles have been estimated at < 2 mol% (29). Techniques for minimizing uncertainties in *T*_mix_ (30) were followed.

**Figure 1:**
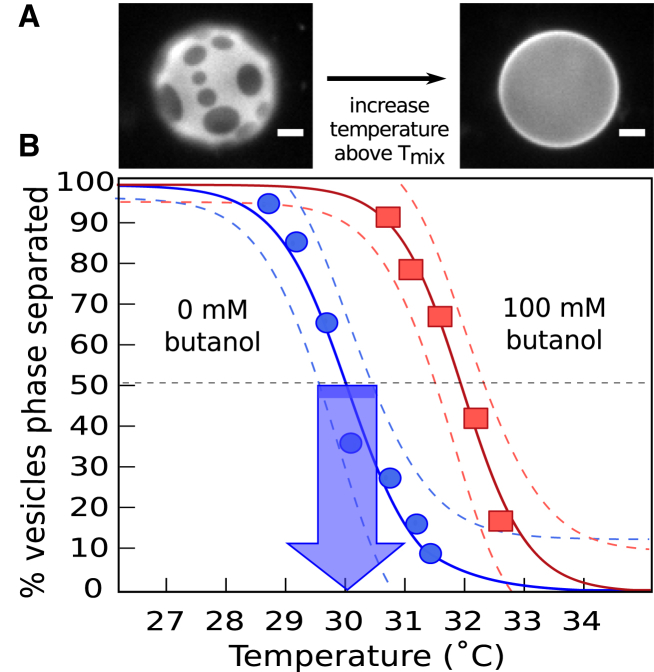
(*A*) Left: Below *T*_mix_, 35/35/30 DOPC/DPPC/cholesterol vesicles exhibit domains of *L*_o_ (*dark*) and *L*_d_ (*bright*) phases. In taut vesicles, domains merge until only one domain of each phase remains (34). Right: Above *T*_mix_, all lipids mix uniformly. Scale bars represent 20 *μ*m. (*B*) *T*_mix_ is higher for 35/35/30 DOPC/DPPC/cholesterol vesicles in 100 mM butanol (*squares*, *T*_mix_ = 32.3 ± 0.19°C) than in pure water (circles, *T*_mix_ = 30.4 ± 0.29°C). Each point records the percent of vesicles with coexisting *L*_o_ and *L*_d_ phases at a given temperature. Dashed lines are 95% confidence intervals (13). The arrow points to *T*_mix_ for the left curve; the width of the arrow’s base is the uncertainty. To see this figure in color, go online.

To image vesicles, several drops of vesicle stock solution were deposited between glass cover slips, and the edges were sealed with vacuum grease. This assembly was thermally coupled to a home-built temperature stage for an epifluorescence microscope (Y-FL; Nikon, Melville, NY) via a layer of thermal grease (Omega Engineering, Stamford, CT). An Alpha-Omega (Lincoln, RI) controller adjusted temperature via a thermoelectric heater/cooler using feedback from a thermistor (0.2°C accuracy; Sensor Scientific, Fairfield, NJ). Images were captured through an air objective using a CoolSnapFX camera (Photometrics, Tucson, AZ) and processed using ImageJ (public domain http://rsbweb.nih.giv/ij). The percent of vesicles that exhibited coexisting *L*_o_ and *L*_d_ phases was recorded over temperature steps between 10 and 50°C. Typically, ∼100 vesicles were imaged at each step, over three fields of view. A nonlinear least squares fit to a sigmoidal curve of % phase separated = 100 × (1–(1/(1 + e^–(*T*–*T*mix)/*B*^))) yields *T*_mix_, at which 50% of vesicles are phase separated, and *B*, which relates to the width of the transition (13). The shift in *T*_mix_, Δ*T*_mix_, is defined as *T*_mix_ (with alcohol) – *T*_mix_ (without alcohol). Uncertainties for each measurement correspond to 95% confidence intervals of the fit, as shown in Fig. 1. For the data in Fig. 1, the dimensionless term Δ*T*_mix_/*T*_mix_ = 0.00626 ± 0.00115 (for temperatures expressed in Kelvin). Uncertainties for a set of measurements due to day-to-day variation is typically < 0.5°C. For example, four experiments on different days using dilute concentrations of (0.05–1 mM) butanol yielded Δ*T*_mix_ of 0.43 ± 0.14°C.

### AC50 values

To compare results with Gray et al. (13), we used AC50 values from Pringle et al. (14): 120 ± 10 mM for ethanol, 54 ± 6 mM for propanol, 12 ± 1 mM for butanol, 0.7 ± 0.1 mM for hexanol, 0.06 ± 0.9 mM for octanol, and 0.013 ± 0.2 mM for decanol. An exponential regression of these values gives 2.96 mM for pentanol. More recent AC50 values by Alifimoff et al. (31) are similar: 190 ± 16 mM for ethanol, 73 ± 2.4 mM for propanol, 10.8 ± 0.77 mM for butanol, 2.9 ± 0.11 mM for pentanol, 0.57 ± 0.37 mM for hexanol, 0.059 ± 0.0031 mM for octanol, and 0.0126 ± 0.00048 mM for decanol. At the AC50, *n*-alcohols are several mol% of membranes (14).

### Cell culture and GPMV isolation

Rat basophilic leukemia (RBL) cells were maintained at 37°C in humidified 5% CO_2_ in growth medium containing 60% modified Eagle’s medium, 30% RPMI medium, 10% fetal calf serum, 2 mM glutamine, 100 units/mL penicillin, and 100 *μ*g/mL streptomycin. GPMVs were isolated and imaged as previously described (32). Briefly, cells were washed in GPMV-buffer (10 mM HEPES, 150 mM NaCl, 2 mM CaCl_2_, pH 7.4) and incubated for 1 h at 37°C in GPMV-buffer supplemented with 25 mM paraformaldehyde and 2 mM dithiothreitol. Differences between results in GUVs and GPMVs are not due to paraformaldehyde and dithiothreitol; miscibility temperatures of GUVs in 18 MΩ-cm water (*T*_mix_ = 31.8 ± 0.15°C, for GUVs of 35:35:30 DOPC/DPPC/cholesterol) are indistinguishable from temperatures of GUVs in 25 mM paraformaldehyde and 2 mM dithiothreitol (*T*_mix_ = 31.9 ± 0.09°C), where uncertainties are determined as in Fig. 1 for single experiments. We verified that our conditions sufficiently replicated the conditions of Gray et al. (13): we found that a short-chain *n*-alcohol (butanol) decreases *T*_mix_ in GPMVs, whereas a long-chain *n*-alcohol (hexadecanol) increases *T*_mix_ (Fig. S6).

### Laurdan and C-laurdan microscopy

GUVs used for laurdan microscopy were electroformed by a method described previously (32) that is functionally equivalent to the method used to produce GUVs for measurements of *T*_mix_. Specifically, 1 *μ*L of a chloroform/methanol (2:1 v/v) solution containing 5 mg/mL of lipids was spread on two platinum electrodes within a Teflon chamber. The lipid mixture contained 0.5 mol% laurdan. The chamber was placed under vacuum for 1 h to dry the solvent and form a lipid film. Next, 350 *μ*L of an aqueous solution (0.2 M sucrose with or without alcohol) was added to the chamber. GUVs were grown by applying an AC voltage of 10 Hz and 2.5 V across the electrodes for 1 h at 52°C. GUVs were then collected and diluted in 1 mL of 0.2 M glucose with or without alcohol.

GUVs and GPMVs were imaged at 20 and 5°C, respectively, by confocal microscopy on a Nikon A1R with spectral imaging at 60× and an excitation of 405 nm. The emission was collected in two bands: 433–463 nm and 473–503 nm. MATLAB (The MathWorks, Natick, MA) was used to calculate two-dimensional general polarization (2D GP) maps, where GP for each pixel was calculated from a ratio of the two fluorescence channels as previously described (33). Briefly, each image was background subtracted and thresholded to retain only pixels with intensities 3–5 standard deviations (SDs) greater than the background in both channels (32). The difference in generalized polarization (ΔGP) between *L*_o_ and *L*_d_ phases was determined for each vesicle, where GP for each phase was derived from average pixel intensities (*I*) from large, representative areas via $GP=\left[\sum_{\mathrm{433}}^{\mathrm{463}}I-\sum_{\mathrm{473}}^{\mathrm{503}}I\right]/\left[\sum_{\mathrm{433}}^{\mathrm{463}}I+\sum_{\mathrm{473}}^{\mathrm{503}}I\right]$.

### Area fractions

Micrographs of vesicle equatorial sections yielded vesicle diameters that were ∼80–250 *μ*m. Videos of the same vesicles were collected such that the top, spherical cap of the vesicle lay inside the < 5 *μ*m depth of field of the microscope objective; the remainder of the vesicle appeared as a bright ring. Centers of vesicles whose bright ring remained in the field of view were identified by custom MATLAB code available by public license in the “Track_Vesicle” program (34, 35). Drift of free-floating vesicles in the *x*-*y* plane was corrected by stacking video frames on vesicle centers. Areas out of focus were excluded, yielding squares with edges ∼15–60 *μ*m. Pixel intensities were thresholded so the *L*_d_ phase was white and the *L*_o_ phase was black. Images within the 2D squares were projected onto 3D spherical surfaces using MATLAB code by Sarah Veatch (36). The area fraction of the *L*_d_ phase was the 3D-projected area of all white pixels divided by the projected area of all pixels in the image.

### High-pressure microscopy

GUVs were electroformed as in the production of GUVs for *T*_mix_ measurements, except that a 0.5 mm PDMS O-ring was used instead of Teflon bars. After electroformation, GUVs were transferred into a custom-built, high-pressure cell mounted on a Nikon Eclipse TE2000-E inverted microscope equipped with a Zyla sCMOS-based camera (Andor Technology, Belfast, UK) (37). The body of the cell was constructed from high-tensile strength stainless steel with openings for 0.5 mm thick, 5 mm square diamond optical windows, which can withstand up to 2500 bar. 1 bar = 10^5^ Pa = 0.987 atm. Hydrostatic pressure was applied via a water-filled pressure generator (4000 bar; SITEC-Sieber Engineering, Maur, Switzerland) and a hydraulic network similar to that described previously (38). Temperature was set at 40°C using a water bath, and images were collected over three fields of view. The pressure was increased from ambient pressure to a maximum of 450 bar, in steps of 50 bar. After each step, the sample was equilibrated for 30 s and images were collected over three fields of view.

## Results and Discussion

### n-Alcohols increase *T*_mix_ in model membranes

When ternary GUVs are formed in aqueous solutions of short-chain *n*-alcohols, the temperatures at which the vesicles demix into coexisting *L*_o_ and *L*_d_ phases increase relative to GUVs in water. For example, in Fig. 1, *T*_mix_ increases by 1.9°C for vesicles of 35/35/30 DOPC/DPPC/cholesterol in 100 mM butanol. This shift is in the opposite direction to that observed in cell-derived GPMVs (13). The difference between the GUV and GPMV results is not due to proteins denaturing, which occurs at butanol concentrations that are roughly an order of magnitude higher (15).

**Figure 2:**
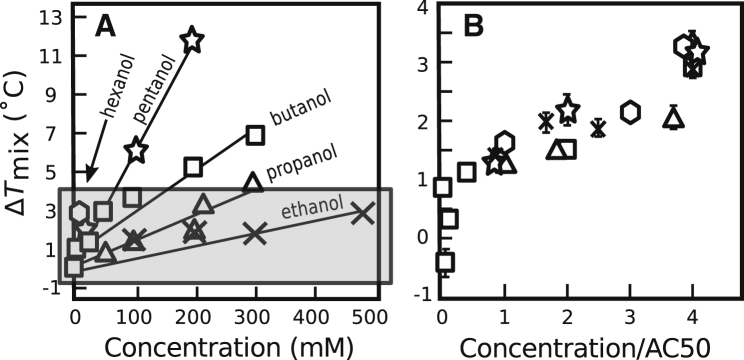
(*A*) Increases in miscibility transition temperatures of 35/35/30 DOPC/DPPC/cholesterol vesicles scale with the concentration of *n*-alcohol in the aqueous solution. Lines are least square fits. (*B*) Data from the shaded region in (A) are rescaled by AC50 values from (14). Because AC50 values are reported to be proportional to partition coefficients of *n*-alcohols from water into PC bilayers (39), concentration/AC50 should be proportional to the concentration of *n*-alcohol in the membrane. Each point represents a single experiment for which symbols are typically larger than uncertainties, determined as in Fig. 1. SDs for repeated experiments are typically ± 0.5°C. Fig. 1 data are not replotted here.

The increase in *T*_mix_ that we observe in model GUVs is robust across a range of short-chain *n*-alcohols and is proportional to the concentration of alcohol in solution (Fig. 2 *A*). When the concentration of each *n*-alcohol is scaled by its AC50 value (14), the data within the shaded area of Fig. 2 *A* collapse (Fig. 2 *B*). The same result holds when AC50 values from (31) are used (Fig. S2). A similar scaling occurs in cell-derived GPMVs (13). Because the AC50 value of an *n*-alcohol is proportional to its partition coefficient from water into PC bilayers (39), the observation of scaling implies that the magnitude of Δ*T*_mix_ is colligative: the value of Δ*T*_mix_ depends only on the mole fraction short chain *n*-alcohol in the membrane.

### Increase in *T*_mix_ is robust across GUV lipid ratios and lipid types

**Figure 3:**
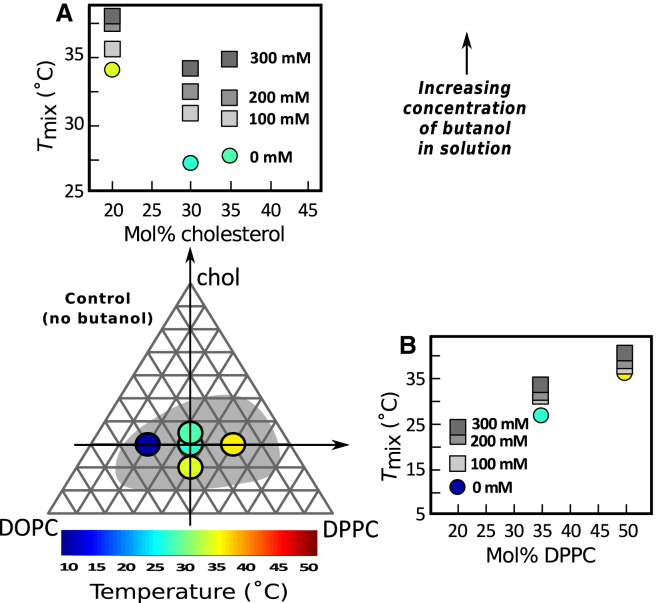
Miscibility transition temperatures of GUVs of five different ratios of DOPC/DPPC/cholesterol increase with the concentration of butanol in solution. Colors within circles on the Gibbs triangle record *T*_mix_ in the absence of butanol; corresponding *T*_*mix*_ values are plotted in (A) and (B). The gray region denotes compositions over which a transition from one uniform phase to coexisting *L*_o_ and *L*_d_ phases is observed at a temperature between 15 and 40°C, from (29). GUVs in (A) contain increasing cholesterol fractions (*following the vertical arrow on the triangle*). GUVs in (B) maintain a constant fraction of cholesterol (*following the horizontal arrow*). Each point represents a single experiment for which uncertainties are smaller than symbols. To see this figure in color, go online.

The result that short-chain *n*-alcohols increase *T*_mix_ by several degrees in model GUV membranes is robust. Fig. 3 shows that this result holds for membranes composed of different ratios of DOPC, DPPC, and cholesterol. The Gibbs triangle in Fig. 3 plots *T*_mix_ values for control GUVs (without butanol) with five different lipid ratios. When GUVs of these same ratios are produced in butanol solutions, *T*_mix_ increases independent of whether the ratios are varied along a vertical (Fig. 3 *A*) or horizontal (Fig. 3 *B*) path in the triangle. A corollary is that short-chain *n*-alcohols increase *T*_mix_ independent of whether the majority of the GUV area is the *L*_o_ phase or by the *L*_d_ phase. Similarly, *T*_mix_ increases independent of whether the GUV membrane is close to or far from a miscibility critical point.

**Figure 4:**
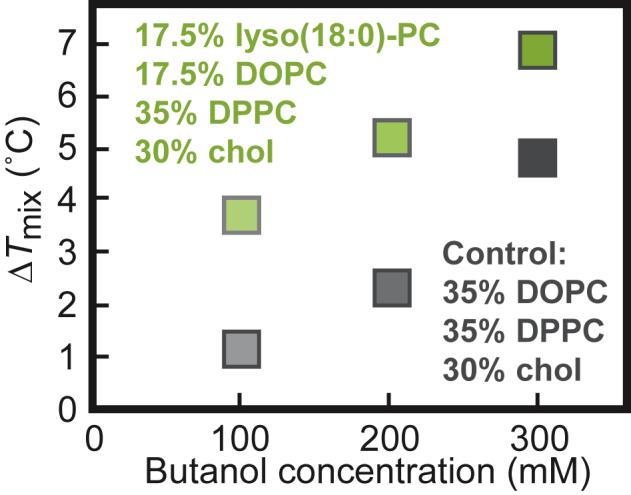
Miscibility transition temperatures of GUVs composed of 17.5/17.5/35/30 lyso(18:0)-PC/DOPC/DPPC/cholesterol (*green*, *top*) and composed of 35/35/30 DOPC/DPPC/cholesterol (*gray, bottom*) increase with the concentration of butanol in solution (increasing darkness of the points). Each point represents a single experiment for which uncertainties are smaller than symbols. Δ*T*_mix_ is with respect to vesicles with no butanol. To see this figure in color, go online.

The result that short-chain *n*-alcohols increase *T*_mix_ is also robust for membranes composed of lipids with different shapes. Lysolipids are cone-shaped lipids with a single acyl tail, which means that lysolipid head groups have larger cross-sectional areas than lysolipid tails. One consequence of this shape is that lysolipids are thought to shield membrane cholesterol, whereas alkanols displace it (40). In Fig. 4, we replace half of the unsaturated DOPC lipids with lyso(18:0)-PC lipids, and we find that butanol still increases *T*_mix_; the change in lipid shape did not change the sign of Δ*T*_mix_.

**Figure 5:**
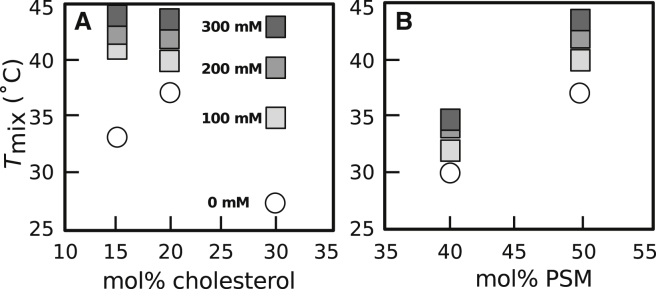
Miscibility transition temperatures for GUVs composed of POPC/PSM/cholesterol increase with the concentration of butanol in solution. (*A*) The molar ratio of POPC to PSM is held constant at 1:1 while the fraction of cholesterol is increased. (*B*) The fraction of cholesterol is held constant at 30 mol% while the ratio of PSM to POPC is increased. Each point represents a single experiment for which uncertainties are smaller than symbols.

The robustness of the result extends to membranes containing lipids with high biological relevance. In Fig. 5, we alter two of the three GUV components. Specifically, we replace DOPC with POPC, and we replace DPPC with PSM. POPC is a common substitute for DOPC because it is ∼18 mol% of PC-lipids in human red blood cells (41). Similarly, PSM constitutes ∼25 mol% of sphingomyelin lipids in red blood cells (41). *T*_mix_ values for control GUVs (without butanol) agree with the previously mapped miscibility phase diagram of POPC/PSM/cholesterol (42, 43). In Fig. 5 *A* and *B*, the ratios of POPC, PSM, and cholesterol are varied in analogy to Fig. 3. For all lipid ratios, *T*_mix_ of GUVs increases with the concentration of butanol in solution.

### Butanol increases *L*_o_-*L*_d_ contrast in GUVs, but not in GPMVs

**Figure 6:**
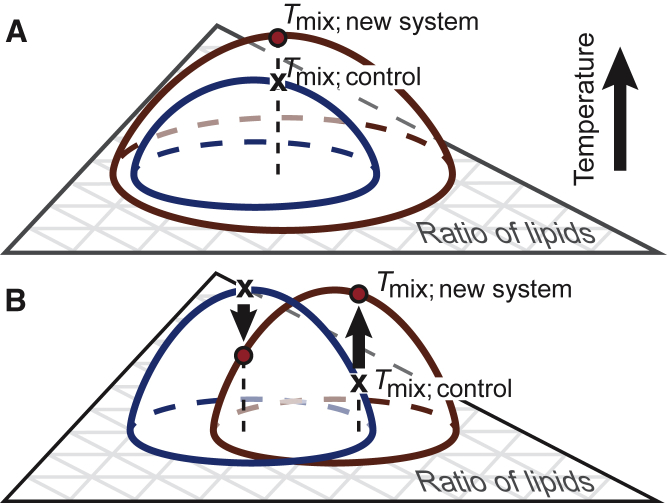
Two scenarios of how miscibility transition temperatures (the curved surfaces) may shift in response to changes in the composition of a membrane. The Gibbs phase triangle in the *x*-*y* plane contains all possible ratios of the three components of the ternary membrane. (*A*) shows an increase in *T*_mix_ over all lipid ratios, upon moving from the control to the new system. (*B*) shows an increase in *T*_mix_ for some lipid ratios (*upward arrow*) and a decrease for others (*downward arrow*). To see this figure in color, go online.

Why does the addition of short-chain *n*-alcohols increase *T*_mix_ in model GUV membranes (Figs. 1, 2, 3, 4, and 5) and decrease *T*_mix_ in cell-derived GPMVs (13)? The simplest explanation is that short-chain *n*-alcohols in GUVs partition much more strongly into one of the membrane phases than the other (44, 45, 46). Given that short-chain alcohols lie directly below lipid headgroups in membranes (1), we expect these alcohols to strongly partition to the *L*_d_ phase of GUVs instead of the *L*_o_ phase. GPMVs are more complex; it is difficult to predict how short-chain alcohols would partition between the *L*_d_ and *L*_o_ phases of a GPMV. If an alcohol were to instead partition roughly equally between the two membrane phases as an “inert diluent,” *T*_mix_ would decrease over all lipid ratios (46, 47). At the very least, we can state that our results in Figs. 3 and 5 are not consistent with the phase boundary merely translating within the plane of the Gibbs phase triangle, such that *T*_mix_ would increase at some lipid ratios and decrease at others. To illustrate these concepts, Fig. 6 *A* shows *T*_mix_ increasing over all lipid ratios, and Fig. 6 *B* shows the phase boundary translating.

Leung and Thewalt (48) found that a particular probe (napthopyrene) partitions strongly to *L*_d_ phases in GUVs, increases the difference in lipid chain order between the two phases, and increases *T*_mix_. They found that an alternate probe (laurdan) partitions weakly between the two membrane phases and does not have these effects (48). If short-chain *n*-alcohols partition strongly to *L*_d_ phases in GUVs and weakly between the two membrane phases in GPMVs, then we would expect short-chain *n*-alcohols to increase the difference in lipid chain order between *L*_o_ and *L*_d_ phases in GUVs and to not increase it substantially in GPMVs. To test this idea, we used the GP of laurdan as a qualitative measure of the difference between *L*_o_ and *L*_d_ phases in GUVs and in GPMVs. Laurdan’s emission spectrum reflects its exposure to aqueous solvent, which in turn reflects the packing of the lipid headgroups, determined in part by the conformational order of lipids in the membrane. Lipid order is a function of membrane composition, and changes in GPMV lipid composition that increase the difference in GP between the *L*_o_ and *L*_d_ phases tend to increase *T*_mix_ of GPMV membranes (32, 48, 49, 50, 51).

**Figure 7:**
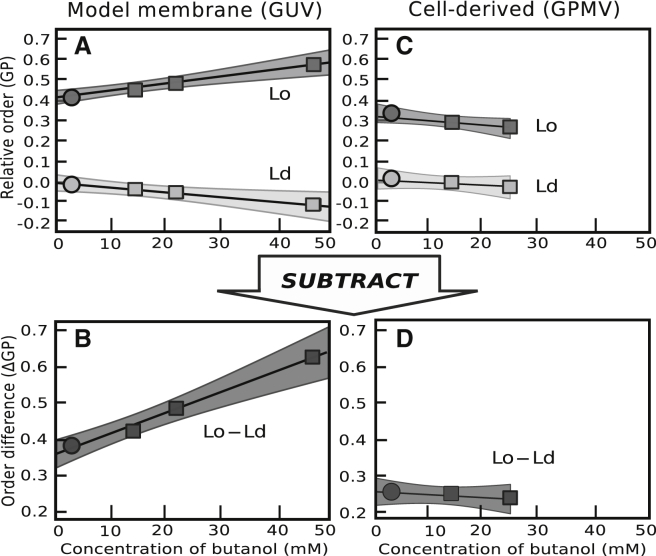
Differences between laurdan GP values in *L*_o_ and *L*_d_ phases in GUVs increase with butanol concentration in solution (*A* and *B*) and remain roughly constant in GPMVs (*C* and *D*). GUVs were composed of 35/35/30 DOPC/DPPC/cholesterol, and GPMVs were derived from RBL cells. Points represent average GP values for batches of 20–30 vesicles on different days. The slope of each line arises from a linear regression with fixed intercepts to offset untreated batch differences from day to day. Shaded areas are 95% confidence intervals of the fit. Slopes of the lines in (*B* and *D*) are 4.80 × 10^−3^ ± 1.37 × 10^−3^ and −6.81 × 10^−4^ ± 1.58 × 10^−3^, in normalized units of ΔGP/[(butanol concentration)(butanol’s membrane-water partition coefficient from (14))]. Fig. S5 and Table S1 contain corresponding data for GUVs and GPMVs with tetradecanol and hexadecanol.

Fig. 7 *A* and *B* show that the addition of butanol, a short-chain *n*-alcohol, does indeed increase the difference in laurdan GP between *L*_o_ and *L*_d_ phases in GUVs, consistent with our expectation that these alcohols strongly partition to the *L*_d_ phase in GUVs. Because short-chain *n*-alcohols generally decrease lipid acyl chain order in model membranes (2, 52, 53, 54, 55, 56), it may seem surprising that incorporation of butanol into the membrane increases the laurdan GP of the *L*_o_ phase in Fig. 7 *A*. However, given that short-chain *n*-alcohols have an antagonistic relationship with cholesterol in membranes (40), *n*-alcohols that partition primarily to the *L*_d_ phase might be expected to drive more cholesterol into the *L*_o_ phase (52, 57, 58), where cholesterol would increase the order of saturated lipids (30, 59). Our results in Fig. 7 *A* and *B* are less consistent with butanol strongly partitioning to the *L*_o_ phase. If most of the butanol partitioned to the *L*_o_ phase, we would expect its laurdan GP to decrease for two reasons: 1) short-chain alcohols decrease lipid chain order (52) and 2) butanol would be expected to displace cholesterol to the *L*_d_ phase, where it would increase the chain order of the lipids in the *L*_d_ phase (60). Instead, the opposite trends in GP values are observed in Fig. 7 *A* and *B*.

Fig. 7 *C* and *D* tell a very different story for cell-derived GPMVs: butanol does not significantly increase the difference in laurdan GP for GPMVs. This result implies that any differential partitioning of butanol between the *L*_o_ and *L*_d_ phases in GPMVs is modest. The contrast between the GUV results in Fig. 7 *A* and *B* and the GPMV results in Fig. 7 *C* and *D* is reminiscent of the contrast between probe partitioning results in GUVs versus GPMVs: in several cases, the same probe partitions strongly between *L*_o_ and *L*_d_ phases in GUVs and weakly between the phases in GPMVs (19, 20, 50, 61).

Theoretically, differential partitioning of butanol between *L*_o_ and *L*_d_ phases in GUVs is measurable from area fractions of the two phases (13). Experimentally, this idea is challenging to test. In control GUVs of 35/35/30 DOPC/DPPC/cholesterol in water, the *L*_d_ phase covers 35.5% of the area (± a SD of 3.4% for n = 18 vesicles). For GUVs of the same lipid composition in 50 mM butanol, the *L*_d_ phase covers 37.2 ± 4.0% for n = 20 vesicles. Within uncertainty, these two values are equivalent. The following estimate shows that even a scenario in which all butanol partitions to one phase would be difficult to resolve within these uncertainties. If we approximate the area per DOPC lipid in a bilayer with 30 mol% cholesterol to be 52.8 Å^2^ (62) and the thickness of a bilayer to be ∼5 nm (63), then every bilayer unit with 100 lipids in each monolayer has a volume of ∼2.6 × 10^−22^ L. If 50 mM butanol in solution partitions into the bilayer with a coefficient of 1.52 as in erythrocytes (14), then each 200-lipid bilayer should harbor ∼12 molecules of butanol, or 6 mol%. Butanol molecules are smaller than lipids. Therefore, with an uncertainty of 4%, we cannot expect to optically resolve area fraction increases due to differential partitioning of butanol.

### Nonmonotonic shifts in *T*_mix_ for long *n*-alcohols

To review, short-chain *n*-alcohols increase membrane *T*_mix_ in GUVs. One of these alcohols, butanol, increases the difference in laurdan GP between the *L*_o_ and *L*_d_ phases of GUVs, presumably as a result of strong preferential partitioning of the butanol to the *L*_d_ phase. As the length of *n*-alcohols increases, we expect a crossover in behavior. Specifically, we expect *n*-alcohols of medium lengths to partition more equally between the *L*_d_ and *L*_o_ phases, resulting in a decrease in *T*_mix_ and no significant increase in ΔGP. Once the number of carbons in *n*-alcohols exceeds a second threshold, we expect the alcohols to again strongly partition to only one of the membrane phases, this time to the *L*_o_ phase. This expectation is reasonable given that a recent calculation predicts that short, saturated alkyl chains partition preferentially to an *L*_d_ phase, that medium chains partition equally, and that long chains partition preferentially to an *L*_o_ phase (64).

**Figure 8:**
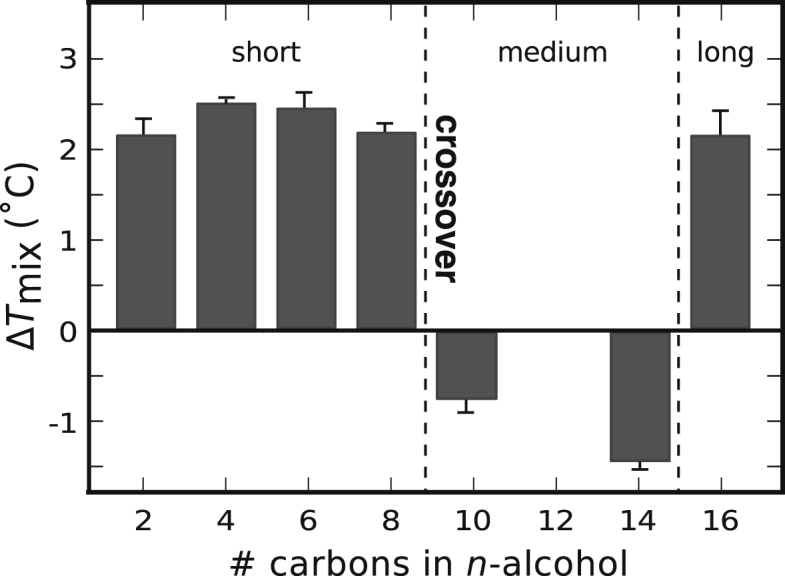
Increasing the number of carbons in *n*-alcohol solutions results in nonmonotonic shifts in *T*_mix_ for GUVs composed of 35/35/30 DOPC/DPPC/cholesterol. Δ*T*_mix_ is with respect to GUVs in water. Concentrations of *n*-alcohol solutions correspond to three times the AC50 in (14). AC50 values for tetradecanol and hexadecanol were estimated at 5 *μ*M. Each point represents a single experiment with uncertainties as in Fig. 1. Fig. S3 contains corresponding data at lower *n*-alcohol concentrations.

Fig. 8 supports the notion that a crossover does indeed occur. Fig. 8 shows that *T*_mix_ increases when GUVs of 35/35/30 DOPC/DPPC/cholesterol are produced in solutions containing *n*-alcohols with short alkyl chains (≤ 8 carbons). In contrast, for medium chains (10–14 carbons), *T*_mix_ decreases. For long chains (≥ 16 carbons), *T*_mix_ increases again. The data in Fig. S5 C are consistent with this view: as a medium chain *n*-alcohol (tetradecanol) is introduced to a GUV solution, there is no significant increase in the difference in laurdan GP between the *L*_o_ and *L*_d_ phases. Recent theory suggests that the greatest decrease in *T*_mix_ should occur when the *n*-alcohol slightly prefers the *L*_d_ phase (M. Schick and D.W. Allender, personal communication).

The designation of *n*-alcohols as “short,” “medium,” and “long” invokes relative terms that depend upon characteristics of the membrane in which the alcohol is embedded. Upon switching from GUVs to GPMVs, *n*-alcohols with 2–10 carbons behave as alcohols in our “medium” category: they decrease *T*_mix_ in GPMVs (11). A length of 16 carbons qualifies as “long” in both types of membranes: in GPMVs, hexadecanol increases *T*_mix_ (45). A switch from “medium” to “long” behavior in GPMVs, where Δ*T*_mix_ ≈ 0, occurs at an alkanol length of 14 carbons (13) (Fig. S4). Addition of tetradecanol to GPMVs results in no significant increase in the difference in laurdan GP values (Fig. S5 D). Gray et al. (13) discuss this switch in behavior as an analogy of the “cutoff effect,” the decrease in efficacy of alcohols as general anesthetics when *n*-alcohols exceed a cutoff length (39, 65).

**Figure 9:**
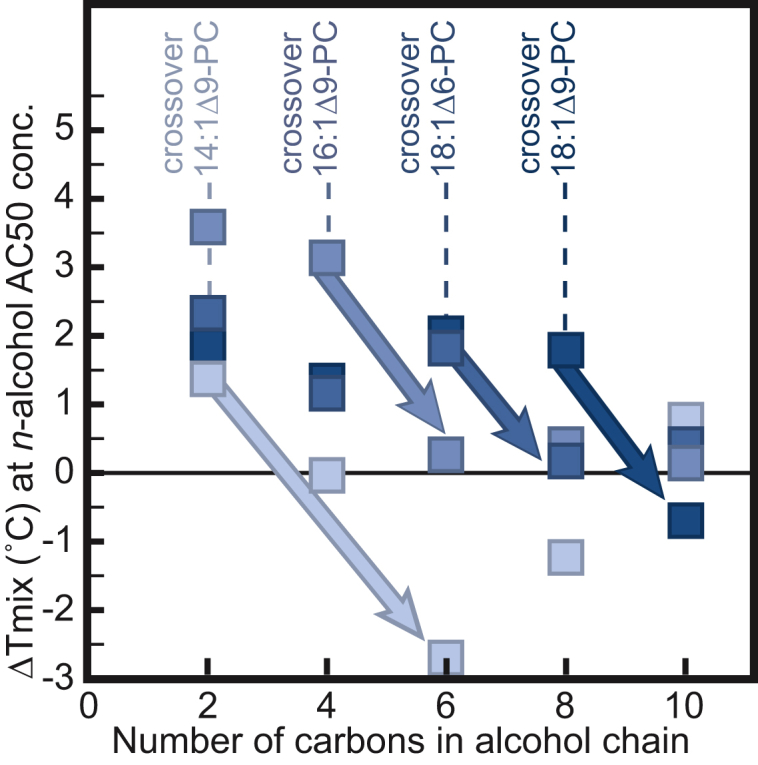
Shift in miscibility transition temperatures for GUVs composed of 35/35/30 mol% mixtures of an unsaturated lipid, DPPC, and cholesterol. Four types of unsaturated lipids were used. For all four, incorporation of an *n*-alcohol with two carbons into the membrane produces a positive Δ*T*_mix_. A significant decrease in *T*_mix_ (> 1°C, shown by *arrows*) occurs at a crossover alkanol length characteristic of each unsaturated lipid. Labeled right to left in the figure, with symbols from darkest to lightest, the unsaturated lipid is either DOPC (18:1 *cis*Δ9 PC), a DOPC analog with the double bond in a different position (18:1 *cis*Δ6 PC), or DOPC analogs with shorter acyl chains (16:1 *cis*Δ9 PC or 14:1 *cis*Δ9 PC). All GUVs were in *n*-alcohol solutions at three times the AC50 concentrations in (14). Δ*T*_mix_ is with respect to vesicles in water. Each point represents a single experiment. Symbols are larger than uncertainties determined as in Fig. 1. To see this figure in color, go online.

Three natural length scales arise in Fig. 8: the length of the alcohol’s alkyl chain, the length of the lipid’s acyl chain, and the length from the lipid’s glycerol backbone to its double bond. These lengths are shown in Fig. S1. In Fig. 9, we decrease the length of *n*-alcohols that qualify as “short” by replacing DOPC with two types of analogous lipids. The first type of replacement uses a lipid with the same length of carbon chain as DOPC and a double bond at a new position (the Δ6 position rather than the Δ9 position). With this substitution, the crossover in behavior occurs at six carbons (i.e., only *n*-alcohols with two, four, or six carbons result in a significant increase *T*_mix_ whereas *n*-alcohols with 8 or 10 carbons do not). The second type of replacement uses lipids with shorter acyl chains, retaining the double bond at the Δ9 position. These new substitutions push the crossover length of the *n*-alcohol down to four carbons (for 16:1Δ9PC lipids) and down to two carbons (for 14:1Δ9PC lipids).

These results imply that the length of the lipids and the position of each lipid’s double bond determine how an *n*-alcohol partitions into *L*_d_ versus *L*_o_ phases. One of the primary differences between GUV and GPMV membranes is in the length and unsaturation of the lipids. Given the high occurrence of polyunsaturated lipids in GPMVs (32), and that *n*-alcohol partition coefficients are highly sensitive to lipid polyunsaturation (66), it is plausible that different *n*-alcohols qualify as “short,” “medium,” and “long” in GPMV membranes than in GUV membranes. It is also plausible that the significant protein content of GPMVs contributes to differences in partitioning of *n*-alcohols between *L*_d_ and *L*_o_ phases of GUVs versus GPMVs. Generalizing from *n*-alcohols to other types of amphiphiles, a long list of molecules are known to partition differently into *L*_d_ versus *L*_o_ phases in GUVs than in GPMVs (18). To highlight the magnitude of this differential partitioning, at least one probe’s preference for the *L*_d_ versus the *L*_o_ phase is reversed in the two types of membranes (19). In summary, differences in how *n*-alcohols partition between the two phases in GUVs and GPMVs may explain why short-chain alcohols increase *T*_mix_ in GUVs and decrease *T*_mix_ in GPMVs.

### Alcohol antiintoxicants increase *T*_mix_ in GUVs

**Figure 10:**
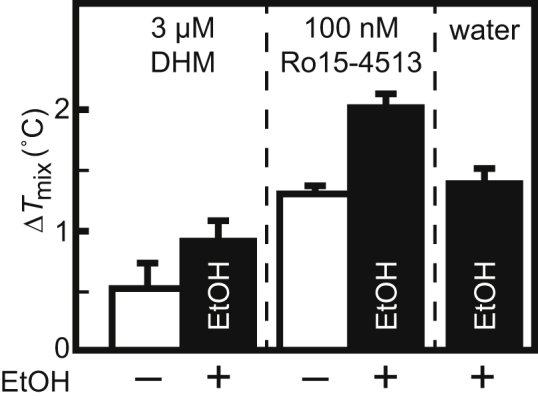
35/35/30 DOPC/DPPC/cholesterol GUVs produced in 3 *μ*M DHM or 100 nM Ro15-4513 (*white bars*) have higher miscibility transition temperatures than control GUVs in water (Δ*T*_mix_). Similarly, GUVs produced in 120 mM ethanol (EtOH, *black bars*) have higher miscibility temperatures under all conditions in the figure. Each bar represents a single experiment for which uncertainties are calculated as in Fig. 1.

If we consider shifts in *T*_mix_ as a consequence of how impurities partition into *L*_o_ and *L*_d_ phases of membranes, we gain a method of predicting whether small molecules will increase or decrease *T*_mix_. Here, we focus on dihydromyricetin (DHM) and Ro15-4513. Both are expected to partition to membranes: Ro15-4513 is roughly twice as hydrophobic as butanol. DHM and Ro15-4513 are termed “antiintoxicants” because they reverse the effects of ethanol in cultured neurons as well as whole organisms, at least at concentrations of 3 *μ*m and 100 nM, respectively (67, 68). These two compounds are also antiintoxicants in terms of reversing the effect of ethanol on *T*_mix_ in cell-derived GPMVs (45). Namely, ethanol decreases *T*_mix_ in GPMVs, whereas DHM and Ro15-4513 increase it (45). The increase in *T*_mix_ implies that DHM and Ro15-4513 partition strongly to only one of the membrane phases in GPMVs. Given that DHM and Ro15-4513 both feature polar groups and bulky ring structures, they are expected to strongly partition near lipid headgroups in the *L*_d_ phase of GUVs and to thereby increase *T*_mix_ in GUVs. Fig. 10 shows that *T*_mix_ of GUVs incubated in 3 *μ*M DHM is indeed higher (by ∼0.5°C) than for GUVs in water. Likewise, Fig. 10 shows that *T*_mix_ for GUVs incubated in 100 nM Ro15-4513 is ∼1.3°C higher than for GUVs in water.

### Minor structural changes in alcohols result in large shifts in membrane *T*_mix_

**Figure 11:**
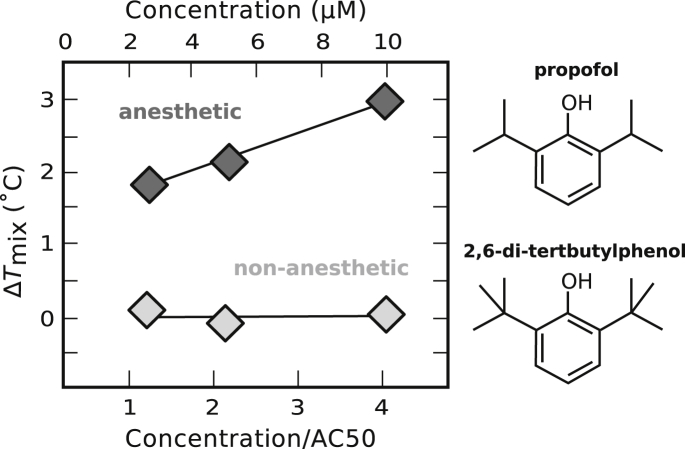
Propofol, a general anesthetic, increases *T*_mix_ in 35/35/30 DOPC/DPPC/cholesterol GUVs. In contrast, 2,6-di-*tert*-butylphenol, which is structurally similar but anesthetically inactive, does not increase *T*_mix_. Each point represents a single experiment. In all cases, symbols are larger than uncertainties determined as in Fig. 1.

In Fig. 10, a cursory description of the bulky, amphiphilic structures of DHM and Ro15-4513 led to a prediction that the compounds would partition strongly into the *L*_d_ phase and increase *T*_mix_. However, partition coefficients are not always straightforward to predict, as for propofol and its membrane-soluble structural analog 2,6-di-*tert*-butylphenol (Fig. 11). This pair is particularly interesting because propofol is a general anesthetic, whereas 2,6-di-*tert*-butylphenol is not (69). Gray et al. (13) previously found that propofol decreases *T*_mix_ in cell-derived GPMVs, whereas 2,6-di-*tert*-butylphenol does not shift *T*_mix_. In other words, propofol behaves as a short-chain alcohol does in GPMVs.

In Fig. 11, we find that propofol also behaves as a short-chain alcohol in GUVs: ternary vesicles in propofol have higher values of *T*_mix_ than vesicles in water. No significant shift in *T*_mix_ occurs for the nonanesthetic analog at identical concentrations. We are unaware of any current models that would predict the partitioning of propofol and 2,6-di-*tert*-butylphenol into *L*_d_ versus *L*_o_ phases. Future models to predict partitioning might incorporate area-to-volume ratios of molecules, as in (70).

### Hydrostatic pressure increases *T*_mix_ in GUVs

Because *T*_mix_ of a GUV membrane is an equilibrium property, it can be tuned by adjusting thermodynamic parameters. For example, *T*_mix_ increases as model vesicles are subjected to increasing hydrostatic pressure (71), indicating that a demixed membrane fills less volume than a uniformly mixed membrane. This result, that *T*_mix_ increases with pressure, holds whether the membrane originates from a model GUV or from a cell-derived GPMV (45).

**Figure 12:**
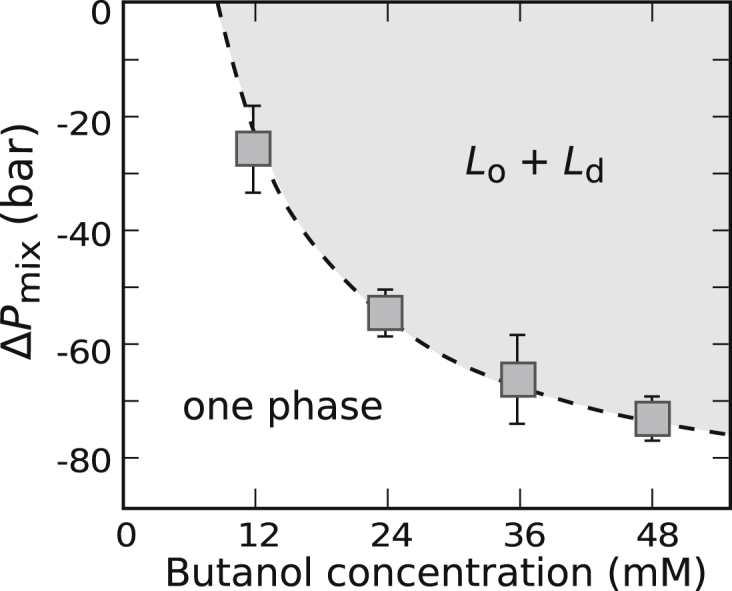
Miscibility transition pressures for GUVs of 35/35/30 DOPC/DPPC/cholesterol decrease with increasing concentration of butanol in solution. Values of Δ*P*_mix_ are relative to control vesicles in water. Each point represents a single experiment for which uncertainties derive from fits to sigmoidal curves of % vesicles separated versus hydrostatic pressure, in analogy to Fig. 1.

Although GUVs and GPMVs are alike in that *T*_mix_ increases with pressure, we have seen in Figs. 1, 2, 3, 4, and 5 that the two systems are dissimilar in their response to short-chain *n*-alcohols. Fig. 12 provides an equivalent illustration of this point. Increasing concentrations of butanol decrease the miscibility transition pressure, *P*_mix_, in model GUVs at constant temperature. These GUV data are consistent with the hypothesis that butanol strongly partitions into only one membrane phase in GUV membranes, such that membranes spontaneously demix over a wider range of conditions. In contrast, in GPMVs, increasing concentrations of butanol increase *P*_mix_ in cell-derived GPMVs (45). Specifically, 12 mM butanol increases *P*_mix_ by 240 ± 30 bar (45). These GPMV data are consistent with the hypothesis that butanol partitions roughly equally into both membrane phases in GPMV membranes (45), such that membranes spontaneously demix over a narrower range of conditions.

### Speculations

In Figs. 1, 2, 3, 4, 5, 6, 7, 8, and 9, we observe shifts in *T*_mix_ and ΔGP that are consistent with 1) short-chain *n*-alcohols partitioning strongly to *L*_d_ phases in ternary GUVs, 2) medium-chain alcohols partitioning roughly equally between *L*_d_ and *L*_o_ phases, and 3) long-chain alcohols partitioning strongly to *L*_o_ phases. In this model, the magnitude of the shift in *T*_mix_ depends on the concentration of *n*-alcohol in a membrane. The experimental question of whether *n*-alcohol concentrations are roughly equivalent in model and cell membranes is not well resolved (39, 72). Our focus on differential partitioning of *n*-alcohols between *L*_o_ and *L*_d_ phases does not exclude other possible modes of action that may differ between GUV and GPMV systems, e.g., *n*-alcohols behaving as lineactants, interacting with proteins, distributing at different distances from the membrane midplane, or competing with cholesterol for phospholipid association.

We speculate that the tendency of an *n*-alcohol to partition differentially between *L*_o_ and *L*_d_ phases depends on characteristic length scales of membrane lipids, set by the location of double bonds and by the length of lipid chains. If this speculation is valid, then GPMVs with different lipid compositions [perhaps due to cell cycle, state, or growth conditions (61, 73)] would have different crossover lengths of *n*-alcohols (see Fig. 9). Similarly, we speculate that any compound that partitions strongly to only the *L*_o_ or *L*_d_ phase will prove to raise *T*_mix_ in membranes. Predicting how subtle structural differences between compounds (e.g., between propofol and 2,6-di-*tert*-butylphenol) are manifested in their partitioning between *L*_o_ and *L*_d_ phases within an all-atom simulation would be expensive given current capabilities. However, the tendency of a compound to strongly preferentially partition to one membrane phase over another may prove to map onto other membrane physical parameters, such as changes in lateral pressure profiles (66) that may be easier to calculate or simulate.

Models that invoke differential partitioning of molecules between *L*_o_ and *L*_d_ phases predict a variety of results. Dilute concentrations of an impurity cause critical temperatures (and hence *T*_mix_) to increase in models that consider differential solubilities (46; M. Schick and D.W. Allender, personal communication) or differential partitioning within an Ising model (44). Experiments in which dilute impurities were added to two-component bulk mixtures found that critical temperatures increased when the impurity had a low solubility in one of the components and decreased when the impurity was likely to be soluble in both components (74). In another model, Schick (75) used the well-known result (76) that for a one-component membrane undergoing a phase transition, an impurity that preferentially partitions into the membrane phase with higher entropy decreases the transition temperature. He showed that a term that relates partitioning of the impurity to the change in transition temperature would also appear in equations describing multi-component membranes. This term, combined with a predicted differential partitioning of alkyl chains (64), could explain why short-chain alcohols decrease *T*_mix_ in GPMVs, but not why they increase *T*_mix_ in GUVs.

## Conclusions

Here we show that *n*-alcohols with ≤ 8 carbons increase *T*_mix_ for membranes of ternary GUV membranes over a range of *n*-alcohol concentrations. This increase is robust for membranes of several lipid types and ratios. As chain lengths of alcohols increase, their effect on membranes is nonmonotonic: *n*-alcohols with 10–14 carbons decrease *T*_mix_ in the GUVs in this study; for *n* = 16, *T*_mix_ increases. Previous experiments using cell-derived GPMVs also found that as chain lengths of alcohols increase, their effect on membranes evolves: *n*-alcohols with ≤ 10 carbons decrease *T*_mix_ in GPMVs; for *n* = 16, *T*_mix_ increases (13, 45). A full summary of how the GUV data compare with previous GPMV data appears in Table S1.

Results from GUVs and GPMVs are equally consistent with a scenario in which the partitioning of *n*-alcohols into *L*_d_ versus *L*_o_ phases changes as the length of the alcohol increases (45). In this scenario, alcohols that are shorter than a characteristic length scale set by the membrane would strongly preferentially partition to the *L*_d_ phase, increasing *T*_mix_. One piece of evidence in support of this scenario is that for GUVs in butanol solutions, laurdan ΔGP values increase with the butanol concentration, as shown in Fig. 7. In contrast, *n*-alcohols of medium length (e.g. tetradecanol, *n* = 14) decrease *T*_mix_ with no significant effect on laurdan ΔGP (Fig. S5).

## Author Contributions

C.E.C., K.R.L., I.L., N.J.B., and S.L.K. designed experiments. C.E.C., K.R.L., I.L., and N.L.C.M. performed experiments. C.E.C. and S.L.K. wrote the manuscript.

## Supporting Material

Document S1. Figs. S1–S6 and Table S1

Document S2. Article plus Supporting Material
