# Supplementary material for: n-Alcohol Length Governs Shift in Lo-Ld Mixing Temperatures in Synthetic and Cell-Derived Membranes
Source: Biophys J. 2017 Aug 9;113(6):1200–11. doi: 10.1016/j.bpj.2017.06.066 (PMC5607138; doi:10.1016/j.bpj.2017.06.066)
Supplement: Document S1. Figs. S1–S6 and Table S1 [file mmc1.pdf]

Biophysical Journal, Volume *113*

Supplemental Information

n-Alcohol Length Governs Shift in L_o-L_d Mixing Temperatures in Synthetic and Cell-Derived Membranes

Caitlin E. Cornell, Nicola L.C. McCarthy, Kandice R. Levental, Ilya Levental, Nicholas J. Brooks, and Sarah L. Keller

SUPPLEMENTARY FIGURES AND TABLES

Lengths of *n*-alcohols govern how L_o-L_d mixing temperatures shift in synthetic and cell-derived membranes

Caitlin E. Cornell[1], Nicola L.C. McCarthy[2], Kandice R. Levental[3], Ilya Levental[3], Nicholas J. Brooks[2], and Sarah L. Keller[1*]

[1]University of Washington, Dept. of Chemistry, Seattle WA 98195 USA
[2]Imperial College London, Dept. of Chemistry, Kensington London SW7 2AZ UK
[3]University of Texas Medical Center, Dept. of Integrative Biology and Pharmacology, Houston TX 77030 USA

[*]Correspondence: slkeller@chem.washington.edu

Table S1. Comparison of GUV and GPMV results.

**GUVs from ternary lipid mixtures (This work)**	**GPMVs derived from RBL cells (References (1) and (2))**	**Figure in main text**
Similarities		
**1.** Short-chain *n*-alcohols cause large shifts in T_{mix} at the AC50 concentration.		Fig. 2
**2.** Shifts in T_{mix} scale with anesthetic dose of short-chain *n*-alcohols.		Fig. 2
**3.** Shifts in T_{mix} diminish when the length of *n*-alcohols reaches a cutoff.		Fig. 8-9
**4.** Long-chain *n*-alcohols (n = 16) increase T_{mix}.		Fig. 8
**5.** Anti-intoxicant compounds (DHM and Ro15-4513) increase T_{mix}.		Fig. 10
**6.** Propofol, a general anesthetic, shifts T_{mix}; its nonanesthetic analog does not.		Fig. 11
Differences		
**1a.** Short *n*-alcohols **increase** T_{mix}.	**1a.** Short *n*-alcohols **decrease** T_{mix}.	Fig. 2
**2a.** Short *n*-alcohols **increase** order differences between L_o and L_d phases (by laurdan generalized polarization)	**2a.** Short *n*-alcohols **do not affect** order differences between L_o and L_d phases (by laurdan generalized polarization)	Fig. 7
**3a.** With increasing concentrations of butanol, P_{mix} **decreases**.	**3a.** With increasing concentrations of butanol, P_{mix} **increases**.	Fig. 12

Figure S1. Names and structures of lipids used in this study. Structures are from (3).

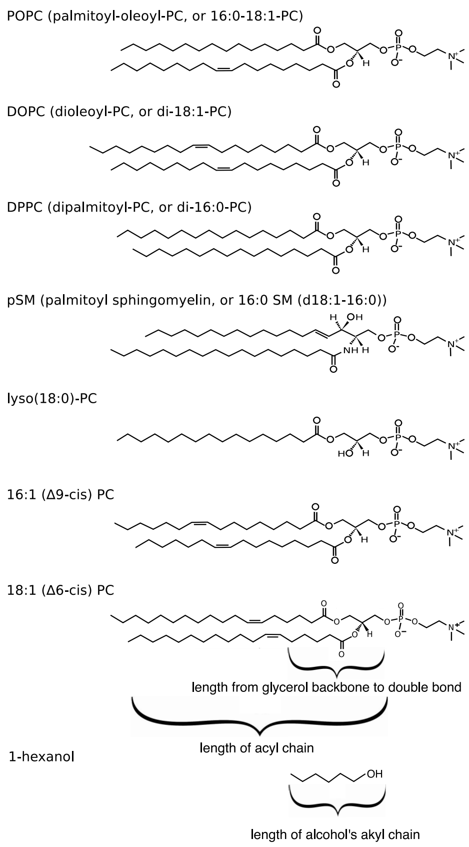

Figure S2. Increases in miscibility transition temperatures of 35/35/30 DOPC/DPPC/chol vesicles scale with *n*-alcohol concentrations up to several times each *n*-alcohol's AC50 value. In Panels A and B, data from Fig. 2a in the main text are rescaled by each *n*-alcohol's AC50 value from reference (4) (Panel A) and from (5) (Panel B). Data diverge at *n*-alcohol concentrations greater than 5 x AC50 (which far exceed physiologically relevant values). Panel C shows data from panel B below 5 x AC50. Symbols are identified in Fig. 2a.

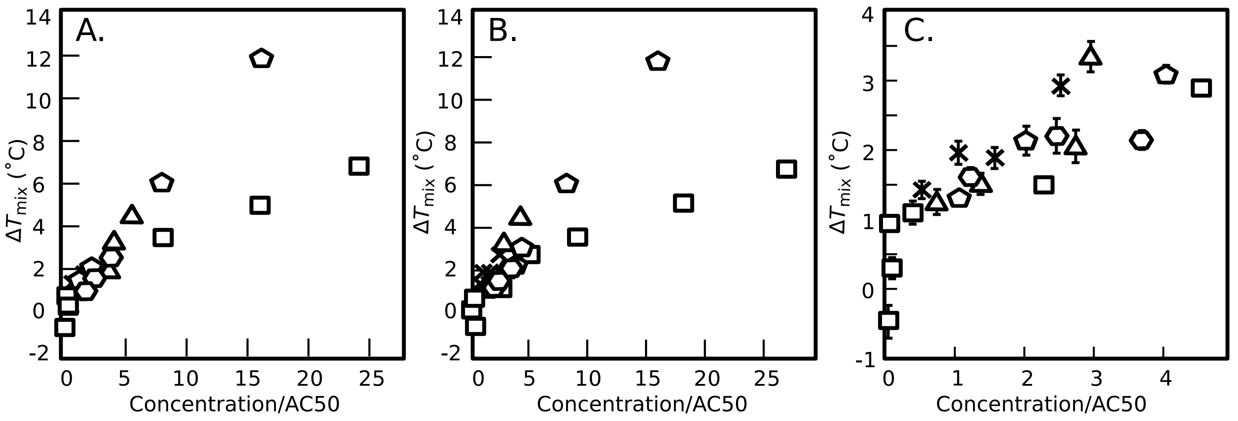

Figure S3. The data in this figure reproduce the data in Figure 8 at two additional AC50 values. In this figure, concentrations of *n*-alcohol solutions are a factor of one (Panel A) and two (Panel B) times the AC50, whereas in Figure 8, concentrations are three times the AC50. For *n*-alcohols of lengths 2-10 carbons, AC50 values were from (4). AC50 values for tetradecanol and hexadecanol were estimated at 5 μM. Increasing the number of carbons in *n*-alcohol solutions results in nonmonotonic shifts in T_{mix} for GUVs composed of 35/35/30 DOPC/DPPC/chol. ΔT_{mix} is calculated with respect to control GUVs in water. Each bar represents a single experiment with uncertainties as in Fig. 1.

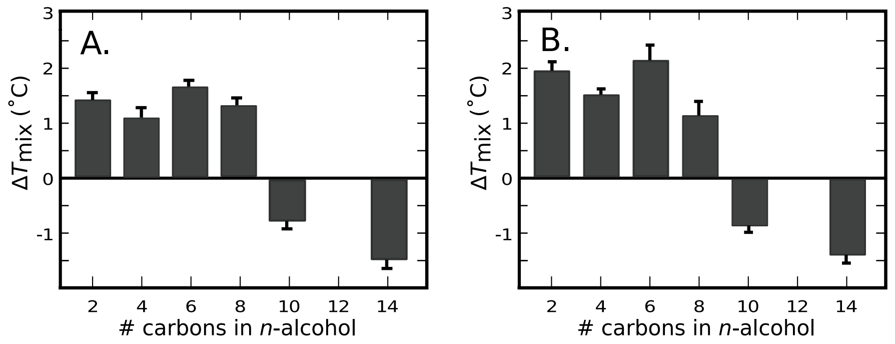

Figure S4. Data from Gray et al. (1) and Machta et al. (2). Increasing the number of carbons in *n*-alcohol solutions results in a crossover in the sign of ΔT_{mix} for GPMVs derived from RBL cells. ΔT_{mix} is calculated with respect to control GPMVs in water. Concentrations of *n*-alcohol solutions are a factor of two times the AC50 for ethanol, octanol, decanol, and hexadecanol and a factor of one times the AC50 for tetradecanol. The value of ΔT_{mix} for hexadecanol was found by assuming additive effects of ethanol and hexadecanol in a GPMV solution containing two times the AC50 of each *n*-alcohol. Data points for ethanol, octanol, decanol, and tetradecanol are extracted from (1) and the data point for hexadecanol is extracted from (2). Each bar represents a single experiment with uncertainties as in Fig. 1.

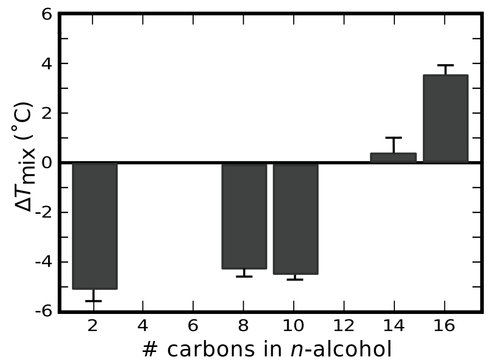

Figure S5. Differences between laurdan GP values in L_o and L_d phases in GUVs and GPMVs remain roughly constant with 1-tetradecanol concentration in solution (panels **A**-**D**). Differences between laurdan GP values in L_o and L_d phases in GUVs and GPMVs slightly increase with 1-hexadecanol concentration in solution (panels **E-H**). GUVs were composed of 35/35/30 DOPC/ DPPC/chol, and GPMVs were derived from RBL cells. Points in the GUV plots represent average GP values for batches of 20-30 vesicles. The slope of each line arises from a linear regression. Points in the GPMV plots represent average GP values for batches of 20-30 vesicles on different days. The slope of each line arises from a linear regression with fixed intercepts to offset untreated batch differences from day to day. Shaded areas are 95% confidence intervals of the fit. Slopes of the lines in Panels C, D, G, and H are $8.94 \times 10^{-7} \pm 2.29 \times 10^{-6}$, $-1.77 \times 10^{-6} \pm 2.80 \times 10^{-6}$, $4.46 \times 10^{-7} \pm 5.82 \times 10^{-7}$, and $4.06 \times 10^{-7} \pm 5.76 \times 10^{-7}$ in units of ΔGP/(butanol concentration x partition coefficient from (4)).

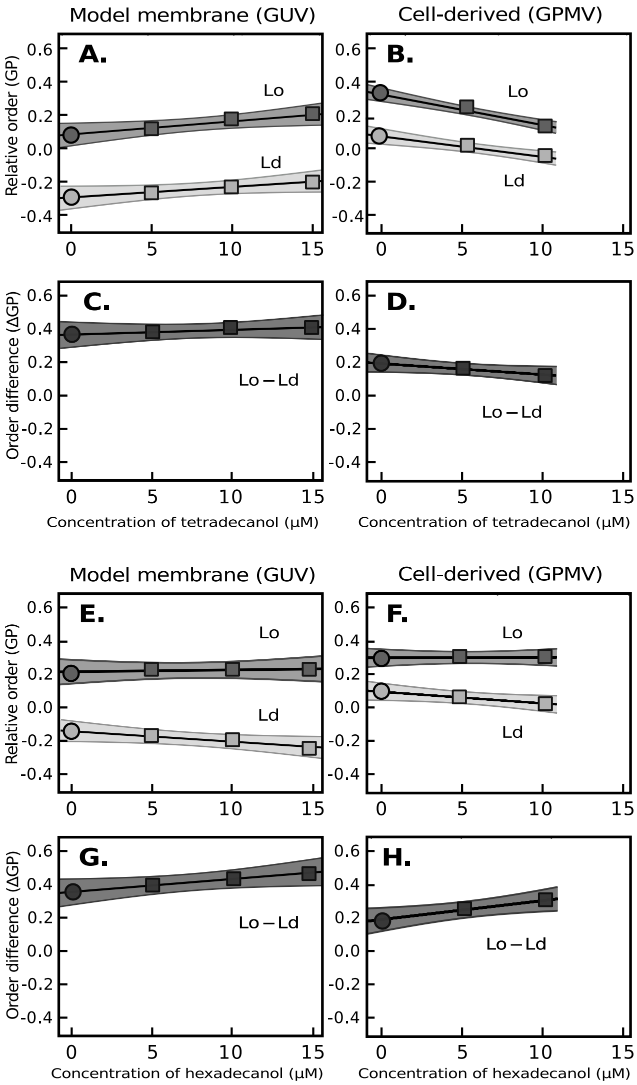

Figure S6. Changes in miscibility transition temperature of GPMVs isolated from RBL cells. **A.** GPMVs incubated in a solution of butanol have a decreased miscibility transition temperature compared to GPMVs incubated in buffer. **B.** GPMVs incubated in a solution of hexadecanol have an increased miscibility transition temperature compared to GPMVs incubated in buffer. Points represent average ΔT_{mix} values over three trials and error bars represent the standard deviation.

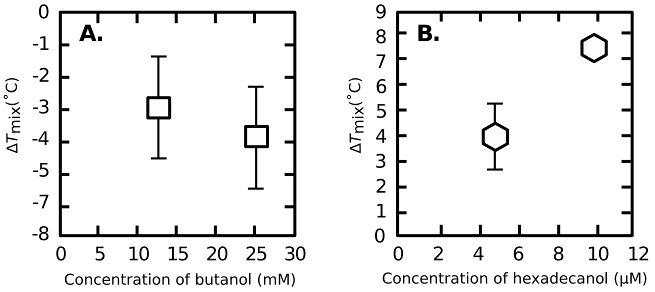

SUPPLEMENTARY REFERENCES

1. Gray, E., J. Karslake, B.B. Machta, and S.L. Veatch. 2013. Liquid general anesthetics lower critical temperatures in plasma membrane vesicles. Biophys. J. 105: 2751–2759.
2. Machta, B.B., E. Gray, M. Nouri, N.L.C. Mccarthy, E.M. Gray, A.L. Miller, N.J. Brooks, and S.L. Veatch. 2016. Conditions that Stabilize Membrane Domains Also Antagonize n-Alcohol Anesthesia. Biophys. J. 111: 537–545.
3. 2017. Avanti Polar Lipids, Inc., http://avantilipids.com.
4. Pringle, M.J., K.B. Brown, and K.W. Miller. 1981. Can the lipid theories of anesthesia account for the cutoff in anesthetic potency in homologous series of alcohols? Mol. Pharmacol. 19: 49–55.
5. Alifimoff, J.K., L.L. Firestone, and K.W. Miller. 1989. Anaesthetic potencies of primary alkanols: implications for the molecular dimensions of the anaesthetic site. Br. J. Pharmacol. 96: 9–16.
